# Brain hyperserotonemia causes autism-relevant social deficits in mice

**DOI:** 10.1186/s13229-018-0243-3

**Published:** 2018-11-26

**Authors:** Miho Tanaka, Atsushi Sato, Shinya Kasai, Yoko Hagino, Hiroko Kotajima-Murakami, Hirofumi Kashii, Yukio Takamatsu, Yasumasa Nishito, Masumi Inagaki, Masashi Mizuguchi, F. Scott Hall, George R. Uhl, Dennis Murphy, Ichiro Sora, Kazutaka Ikeda

**Affiliations:** 1 Department of Psychiatry and Behavioral Sciences, Addictive Substance Project, Tokyo Metropolitan Institute of Medical Science, 2-1-6 Kamikitazawa, Setagaya-ku, Tokyo, 156-8506 Japan; 2 Molecular and Cellular Medicine, Graduate School of Medical and Dental Sciences, Niigata University, Niigata, Japan; 3 Department of Developmental Disorders, National Institute of Mental Health, National Center of Neurology and Psychiatry, Tokyo, Japan; 4 Department of Pediatrics, The University of Tokyo Hospital, Tokyo, Japan; 5 Center for Basic Technology Research, Tokyo Metropolitan Institute of Medical Science, Tokyo, Japan; 6 Department of Developmental Medical Sciences, Graduate School of Medicine, The University of Tokyo, Tokyo, Japan; 7 Department of Pharmacology and Experimental Therapeutics, College of Pharmacy and Pharmaceutical Sciences, The University of Toledo, Toledo, OH USA; 8 Branch of Molecular Neurobiology, National Institute on Drug Abuse, Baltimore, MD USA; 9 Research Service, New Mexico VA Health Care System, Albuquerque, NM USA; 10 Laboratory of Clinical Science, National Institutes of Health, Bethesda, MD USA; 11 Department of Psychiatry, Kobe University Graduate School of Medicine, Kobe, Japan

**Keywords:** Serotonin transporter, Tryptophan depletion, Autism spectrum disorder, Heterozygous mice

## Abstract

**Background:**

Hyperserotonemia in the brain is suspected to be an endophenotype of autism spectrum disorder (ASD). Reducing serotonin levels in the brain through modulation of serotonin transporter function may improve ASD symptoms.

**Methods:**

We analyzed behavior and gene expression to unveil the causal mechanism of ASD-relevant social deficits using serotonin transporter (*Sert*) knockout mice.

**Results:**

Social deficits were observed in both heterozygous knockout mice (HZ) and homozygous knockout mice (KO), but increases in general anxiety were only observed in KO mice. Two weeks of dietary restriction of the serotonin precursor tryptophan ameliorated both brain hyperserotonemia and ASD-relevant social deficits in *Sert* HZ and KO mice. The expression of rather distinct sets of genes was altered in *Sert* HZ and KO mice, and a substantial portion of these genes was also affected by tryptophan depletion. Tryptophan depletion in *Sert* HZ and KO mice was associated with alterations in the expression of genes involved in signal transduction pathways initiated by changes in extracellular serotonin or melatonin, a derivative of serotonin. Only expression of the *AU015836* gene was altered in both *Sert* HZ and KO mice. *AU015836* expression and ASD-relevant social deficits normalized after dietary tryptophan restriction.

**Conclusions:**

These findings reveal a *Sert* gene dose-dependent effect on brain hyperserotonemia and related behavioral sequelae in ASD and a possible therapeutic target to normalize brain hyperserotonemia and ASD-relevant social deficits.

**Electronic supplementary material:**

The online version of this article (10.1186/s13229-018-0243-3) contains supplementary material, which is available to authorized users.

## Background

Autism spectrum disorder (ASD) is a neurodevelopmental disorder that has two symptomatic domains: (1) deficits in verbal and nonverbal social communication and reciprocal social interaction and (2) restricted, repetitive patterns of behavior, interests, and activity [1]. The prevalence of ASD was previously reported to be less than 0.1% [2–5], but ASD is now found in more than 1% of the general population [6]. The pathogenesis of ASD is multifactorial. More than 400 genes and more than 40 genetic loci have been shown to be associated with ASD, including genes associated with the function of neurotransmitter serotonin (5-hydroxytryptamine [5-HT]) [2, 3, 5].

The relationship between ASD and abnormal 5-HT metabolism has been recognized for decades. Elevations of 5-HT levels in whole blood and platelets are detected in approximately 30% of individuals with ASD with or without intellectual disability [7–9]. Elevated 5-HT levels in platelets are also observed in individuals with ASD without intellectual disability [10]. This pattern of alterations in 5-HT metabolism may involve a decrease in the function of the serotonin transporter (SERT). The *SLC6A4* gene encodes SERT at chromosomal region 17q11, a major susceptibility locus in ASD [11]. Individuals with the short allele of the *SLC6A4* gene-linked polymorphic region (5-HTTLPR) are more likely to present with greater anxiety, impairments in social interaction, and deficits in emotional regulation [12]. The short allele of 5-HTTLPR is associated with a decrease in SERT expression [13] and alterations of amygdala function in ASD [14]. Lower SERT binding affinity is also found in the brains of adult individuals with ASD [15]. This evidence strongly suggests that there is a close link between ASD and low SERT expression.

The neuropsychiatric effects of high 5-HT levels have been investigated in *Sert* knockout (KO) mice with no *Sert* expression. *Sert* KO mice exhibit high levels of 5-HT in the brain [16–18] and high 5-HT terminal density in the neocortex [19]. However, elevations in extracellular 5-HT levels in *Sert* KO mice produce compensatory reductions in other aspects of 5-HT function, including reduced 5-HT synthesis and tissue content [20]. Behavioral alterations in *Sert* KO mice include impaired locomotor function, increased anxiety, and reduced aggression [21–23]. Heterozygous (HZ) deletion of the *Sert* gene also affects stress-induced behavior in the forced swim test [22]. However, rodent models of SERT deletion show inconsistent results for behaviors relevant to ASD [24–26], prompting further investigation.

Serotonin is synthesized from the essential amino acid tryptophan by tryptophan hydroxylase (TPH). Dietary tryptophan restriction effectively reduces intra- and extracellular 5-HT levels in the brain and has been used to investigate the involvement of 5-HT in diverse brain functions [27, 28]. A tryptophan-free diet lowers the ability to recognize faces expressing fear or happiness [29, 30]. In individuals with ASD, tryptophan depletion lowers plasma tryptophan levels and aggravates stereotyped behavior [30, 31]. At the same time, abnormal brain connectivity in ASD, involving the cerebral cortex, basal ganglia, and cerebellum, is improved by a tryptophan-restricted diet. This evidence suggests an underlying influence of low brain 5-HT levels in some ASD deficits [32]. Moreover, since altering 5-HT function was beneficial in adults and did not require treatment during development, it encourages exploration of treatment approaches that seek to normalize 5-HT function later in life.

Also supporting this potential role for alterations in 5-HT metabolism in ASD are studies in tryptophan hydroxylase 2 (*Tph2*) KO mice that have greatly reduced 5-HT levels in the brain (~ 96% reduction) [33, 34] and exhibit ASD-like behavioral deficits [35, 36]. In a non-genetic model, early life depletion of 5-HT with 5,7-dihydroxytryptamine also produces autism-like phenotypes [37]. The levels of tissue 5-HT depletion in these models are more severe than those in the *Sert* KO mice. Although *Sert* KO mice exhibit behavioral changes associated, or commonly comorbid, with ASD, it remains to be established whether *Sert* KO mice demonstrate social deficits characteristic of ASD. In addition, it might be hypothesized that elevating brain 5-HT levels might alleviate ASD-related behavioral deficits in these mice.

In the present study, ASD-relevant social deficits were observed in *Sert* HZ and KO mice, and such deficits were rescued by 2 weeks of a tryptophan-depleted diet, which lowered brain 5-HT levels and normalized the expression of some genes within the 5-HT system. The findings in this model implicate a causal role for high brain 5-HT levels in the pathogenesis of ASD.

## Methods

### Animals

*Sert* KO mice were generated as previously described [20] and backcrossed onto a C57BL/6 J genetic background for eight generations [38]. Wild-type (WT), *Sert* HZ, and *Sert* KO littermates were obtained by crossing male HZ and female HZ mice. Both male and female mice of the three genotypes were used. Mice were housed in groups of three to six littermates per cage and maintained on a 12-h/12-h light/dark cycle, with free access to food and water. Naive mice were tested between 3 and 6 months of age. They were examined during the light phase of the light/dark cycle in an experimental room under white light conditions. The first cohort was tested in the elevated plus maze, the hole-board test, the social interaction test, and the three-chamber test. The experiments were conducted at intervals of 1 week or longer. Mice from different cohorts were used in the elevated plus maze test because of the adjusted light conditions (Additional file 1: Table S1). Mice from the second cohort were allocated to either the tryptophan-free diet or a control diet, and locomotion and social interaction were examined. The third cohort was used to assess the effects of the *Sert* genotype and tryptophan-free diet on extracellular 5-HT levels using microdialysis. The fourth cohort was used for gene expression analyses. In these mice, 10 litters were divided into six groups (Additional file 1: Table S2). Mice were cared for and treated humanely in accordance with all institutional and national animal experimentation guidelines.

### Intervention with tryptophan-free diet

A tryptophan-free diet (Trp−, Oriental Yeast Company, Tokyo, Japan) was used to examine the influence of reducing tryptophan availability on perturbations in brain 5-HT function resulting from reduced SERT function. Mice of all genotypes were divided into two groups: one group received Trp− for 2 weeks, and the other group received a control diet (Ctrl) that contained normal levels of dietary tryptophan for 2 weeks. The mice underwent the locomotor test and social interaction test after 7 and 14 days on the diet, respectively. Microdialysis and brain sampling for gene expression analysis were also conducted after 14 days on the diet.

### Behavioral tests

#### Social interaction test

The mice were left alone in the home cage for 15 min for habituation, after which a novel C57BL/6 J mouse of the same sex was introduced into the cage. The 10-min test was digitally recorded, and the duration of active social interaction (i.e., sniffing, allogrooming, mounting, and chasing of the tested mouse toward the novel mouse) was determined by observers who were blind to the treatment conditions and genotype [39].

#### Three-chamber test

The apparatus consisted of an open-topped acrylic box (500 mm × 500 mm × 400 mm) divided into three chambers. The test consisted of three phases: habituation, social approach (stranger mouse 1 [S1] vs. inanimate object [Ob]), and social preference (S1 vs. stranger mouse 2 [S2]). The test mouse was first placed in the middle chamber and allowed to freely explore the empty apparatus for 10 min. A novel C57BL/6 J mouse of the same sex (S1) and the Ob (i.e., an aluminum cylinder; 30 mm radius, 60 mm height) were placed in a small wire cage in the left and right compartments for 10 min. Afterward, Ob was replaced with S2, and the test mouse was allowed another 10 min of free exploration in the social preference phase. The sides where Ob, S1, and S2 were placed were randomly assigned. The time spent exploring each cage was measured using a video tracking system in all phases (Muromachi Kikai, Tokyo, Japan).

#### Elevated plus maze

The apparatus consisted of two open arms (297 mm × 54 mm) and two closed arms (300 mm × 60 mm, with 150-mm-high walls) that were set in a plus configuration. The apparatus was raised 400 mm above the floor. The test mouse was allowed to freely explore the apparatus for 10 min. The time on the open arms, number of entries into the open and closed arms, and total distance traveled were recorded by a video tracking system (Muromachi Kikai).

#### Hole-board test

The apparatus consisted of a field (500 mm × 500 mm × 400 mm) that had four holes (30-mm diameter each). The test mouse was allowed to freely explore the apparatus. The total duration and number of head dips into the holes were recorded for 30 min using a video tracking system (Muromachi Kikai).

#### Locomotor activity test

The apparatus consisted of an illuminated chamber (300 mm × 400 mm × 250 mm). Each mouse was left alone in the apparatus, and total locomotor activity was measured for 60 min using a Supermex system (Muromachi Kikai), with a sensor monitor mounted above the chamber.

### Microdialysis

The mice were implanted with microdialysis probes in the striatum (anterior, + 0.6 mm; lateral, + 1.8 mm; ventral, − 4.0 mm from bregma) after anesthesia with sodium pentobarbital (50 mg/kg, intraperitoneal). At 24 h after surgery, the levels of 5-HT were measured using microdialysis under freely moving conditions. Mice were dialyzed with Ringer’s solution (145 mM NaCl, 3 mM KCl, 1.26 mM CaCl_2_, and 1 mM MgCl_2_, pH 6.5) at a flow rate of 1 μl/min. Data were collected every 10 min for 180 min, and perfusion was initiated 180 min before the collection of baseline samples. 5-HT in the dialysates was separated using a reverse-phase ODS column (PP-ODS, Eicom, Kyoto, Japan) and detected with a graphite electrode (HTEC-500, Eicom). The mobile phase consisted of 0.1 M phosphate buffer (pH 5.5) that contained sodium decanesulfonate (500 mg/l), ethylenediaminetetraacetic acid (EDTA; 50 mg/l), and 1% ethanol.

### Whole-genome gene expression analysis

Whole-genome gene expression profiles were analyzed using the Mouse Gene Expression 4x44K v2 Microarray (Agilent Technologies, Tokyo, Japan), which detects 39,430 Entrez Gene RNAs. Total RNA was first isolated with TRIzol reagent (ThermoFisher Scientific, Waltham, MA, USA) from whole brains of four male mice in each group with the Ctrl or Trp− diet. Total RNA was determined using an Agilent 2100 Bioanalyzer. Total RNA was then applied to Cy3-labeled cRNA synthesis, which was performed with a Low Input Quick Amp Labeling Kit (Agilent Technologies) according to the manufacturer’s instructions. Finally, Cy3-labeled cRNA was hybridized to the microarray and detected using an Agilent SureScan Microarray Scanner (Agilent Technologies).

Microarray image data were extracted to ProcessedSignal using Feature Extraction 11.5.1.1 software (Agilent Technologies). GeneSpring GX v14.5 software (Agilent Technologies) was used for subsequent data processing. Gene expression with a statistically significant difference (*P* < 0.05) and an absolute value of log_2_ fold change > 1.2 between groups was exported to the dataset. Gene ontology and pathway analyses were performed using BaseSpace (Illumina KK, Tokyo, Japan) and MetaCore v6.30 build 68780 (Clarivate Analytics Japan, Tokyo, Japan), respectively.

### Statistical analysis

The statistical analyses were performed with Excel Statistics software (Microsoft Japan, Tokyo, Japan). The behavioral data were analyzed using analysis of variance (ANOVA; two-way repeated measures) followed by Fisher’s PLSD post hoc test and Student’s *t* test. For the gene expression analysis, an unpaired *t* test was performed among genotypes and treated groups. Values of *P* < 0.05 were considered statistically significant.

## Results

### *Sert* HZ and KO mice exhibit aberrant social interaction

**Fig. 1:**
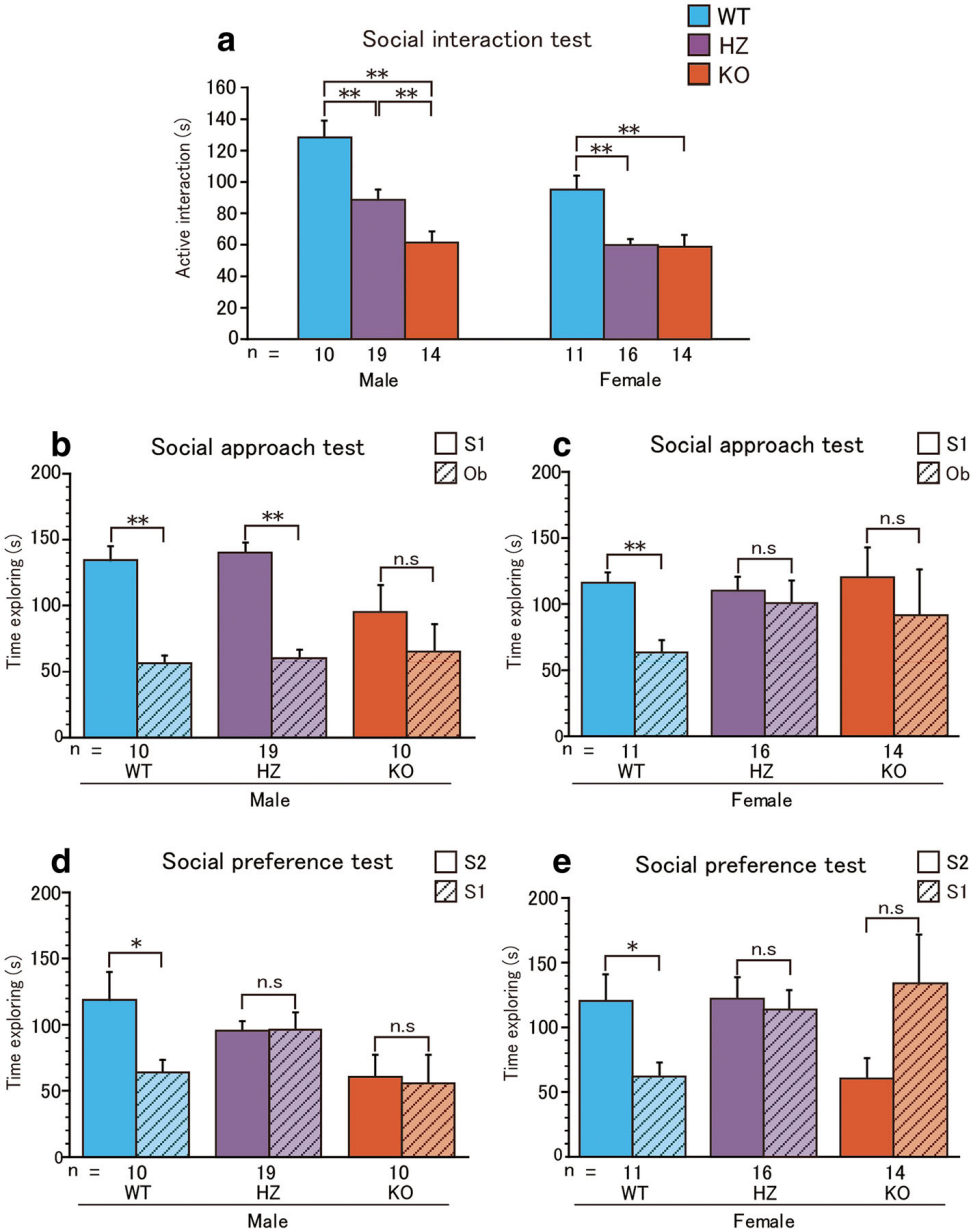
Expression of ASD-relevant social deficits in *Sert* HZ and KO mice. **a** Active interaction time over 10 min in the social interaction test. The data are expressed as mean ± SEM. *P < 0.05, **P < 0.01, two-way ANOVA followed by Fisher’s PLSD test. **b**, **c** Active social interaction time for 10 min in the social approach phase in the three-chamber test. **d**, **e** Active social interaction time over 10 min in the social preference phase in the three-chamber test. The data are expressed as mean ± SEM. *P < 0.05, **P < 0.01, paired Student’s *t* test (**b**–**e**)

In the social interaction test (Fig. 1a), active social interaction was affected by genotype (*F*_2,78_ = 22.98, *P* < 0.001) and sex (*F*_1,78_ = 12.80, *P* < 0.001), but there was no significant interaction between these factors (*F*_2,78_ = 2.51, *P* = 0.087). Active social interaction was reduced in both male and female *Sert* HZ and KO mice compared to WT mice as confirmed by post hoc comparisons (male *Sert* HZ < WT mice, *P* < 0.001; male *Sert* KO mice < WT and *Sert* HZ mice, *P* < 0.001 [WT], *P* = 0.0054 [HZ]; female *Sert* HZ and KO < WT mice, *P* = 0.0014 [WT vs. HZ], *P* = 0.0013 [WT vs. KO]).

In the three-chamber test of social preference (Fig. 1b, c), both WT and male *Sert* HZ mice exhibited intact social approach that was greater toward S1 compared with Ob (*t*_9_ = 6.15, *P* < 0.001 [male WT], *t*_18_ = 8.19, *P* < 0.001 [male HZ], *t*_10_ = 3.67, *P* = 0.0042 [female WT] by paired *t* test). Male *Sert* KO, female HZ, and female KO mice did not show a social preference, spending a comparable time exploring S1 and Ob (*t*_9_ = 1.11, *P* = 0.29 [male KO], *t*_15_ = 0.38, *P* = 0.70 [female HZ], *t*_13_ = 0.59, *P* = 0.55 [female KO] by paired *t* test). In the social novelty phase of the experiment (Fig. 1d, e), WT mice had normal social novelty preference (*t*_9_ = 2.72, *P* = 0.023 [male WT], *t*_10_ = 2.29, *P* = 0.044 [female WT] by paired *t* test). *Sert* HZ and KO mice did not differentiate S2 from S1 (*t*_18_ = − 0.11, *P* = 0.91 [male HZ], *t*_9_ = 0.22, *P* = 0.82 [male KO], *t*_15_ = 0.33, *P* = 0.74 [female HZ], *t*_13_ = − 1.67, *P* = 0.11 [female KO] by paired *t* test). These results suggest that HZ *Sert* deletion is sufficient to influence social behavior in mice.

### *Sert* KO mice exhibit enhanced anxiety-like behavior

**Fig. 2:**
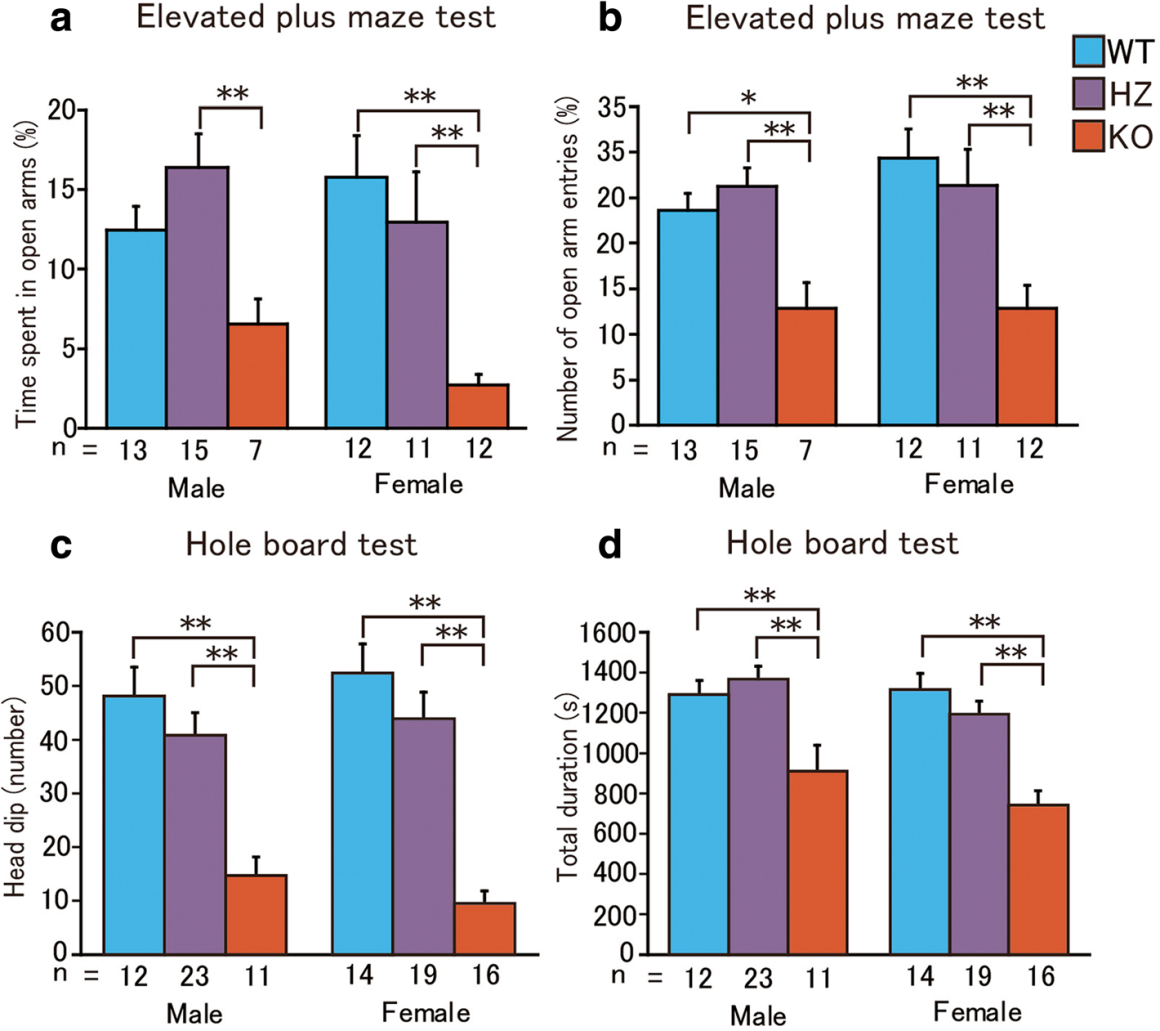
Anxiety-related behavior in *Sert* KO mice. **a** Time spent on the open arms in the elevated plus maze. **b** Number of open arm entries in the elevated plus maze. **c** Number of head dips in the hole-board test. **d** Total distance traveled in the hole-board test. The data are expressed as mean ± SEM. *P < 0.05, **P < 0.01, two-way ANOVA followed by Fisher’s PLSD test

We next assessed anxiety-like behavior because a heightened level of general anxiety (rather than social anxiety per se) may impair response to novel social stimuli. In the elevated plus maze test (Fig. 2a, b), *Sert* KO mice exhibited an increase in anxiety-like behavior, reflected by decreases in the percent time on the open arms and number of open arm entries. The ANOVA revealed significant effects of genotype on the time spent on the open arms (*F*_2,64_ = 11.77, *P* < 0.001) and number of open arm entries (*F*_2,64_ = 13.37, *P* < 0.001), with no effect of sex (time spent on open arms: *F*_1,64_ = 0.52, *P* = 0.46; open arm entries: *F*_1,64_ = 0.68, *P* = 0.41) and no genotype × sex interaction (time spent on open arms: *F*_2,64_ = 1.79, *P* = 0.17; open arm entries: *F*_2,64_ = 0.71, *P* = 0.49). Male *Sert* KO mice also exhibited a decrease in the percent time on the open arms compared with *Sert* HZ mice (*P* = 0.0044 [HZ vs. KO] by Fisher’s PLSD post hoc test) but not with WT mice (*P* = 0.089 [WT vs. KO] by Fisher’s PLSD post hoc test). Female *Sert* KO mice exhibited a decrease in the percent time on the open arms compared with WT and *Sert* HZ mice (*P* < 0.001 [WT vs. KO], *P* = 0.0013 [HZ vs. KO] by Fisher’s PLSD post hoc test). *Sert* HZ mice were comparable to WT mice for both males and females (male: *P* = 0.15 [WT vs. HZ]; female: *P* = 0.35 [WT vs. HZ] by Fisher’s PLSD post hoc test). The number of open arm entries was lower in *Sert* KO mice compared with WT and *Sert* HZ mice (male: *P* = 0.018 [WT vs. KO], *P* = 0.0030 [HZ vs. KO]; female: *P* < 0.001 [WT vs. KO], *P* = 0.0011 [HZ vs. KO] by Fisher’s PLSD post hoc test). *Sert* HZ mice were comparable to WT mice for both males and females (male: *P* = 0.47 [WT vs. HZ]; female: *P* = 0.44 [WT vs. HZ] by Fisher’s PLSD post hoc test).

*Sert* KO mice also made fewer head dips in the hole-board test (Fig. 2c, d), as shown by a significant effect of genotype (*F*_2,89_ = 35.93, *P* < 0.001), but not sex (*F*_1,89_ = 0.029, *P* = 0.86). There was also no significant interaction of genotype and sex (*F*_2,89_ = 0.58, *P* = 0.56). *Sert* KO mice were again different from both WT and *Sert* HZ mice of both sexes (male: *P* < 0.001 [WT vs. KO], *P* < 0.001 [HZ vs. KO]; female: *P* < 0.001 [WT vs. KO], *P* < 0.001 [HZ vs. KO]). Again, *Sert* HZ mice were not different from WT mice (*P* = 0.23 [male], *P* = 0.16 [female]). The total duration in the hole-board test was also affected by genotype (*F*_2,89_ = 22.65, *P* < 0.001) but not by sex (*F*_1,89_ = 2.65, *P* = 0.10), with no genotype × sex interaction (*F*_2,89_ = 1.03, *P* = 0.36). The total duration in the hole-board test was shorter in *Sert* KO mice than in WT and *Sert* HZ mice (male: *P* = 0.0032 [WT vs. KO], *P* < 0.001 [HZ vs. KO]; female: *P* < 0.001 [WT vs. KO], *P* < 0.001 [HZ vs. KO] by Fisher’s PLSD post hoc test). The total duration was comparable between WT and *Sert* HZ mice of both sexes (*P* = 0.47 [male], *P* = 0.21 [female] by Fisher’s PLSD post hoc test).

In summary, an increase in general anxiety-like behavior was observed in *Sert* KO mice but not in *Sert* HZ mice. *Sert* HZ mice exhibited abnormal social behavior in different tasks. These findings suggest that ASD-relevant social deficits may be caused by low *SERT* expression, independent of overall changes in anxiety.

### Tryptophan-free diet reduces extracellular 5-HT in the striatum and improves ASD-relevant social deficits in *Sert* mutant mice

**Fig. 3:**
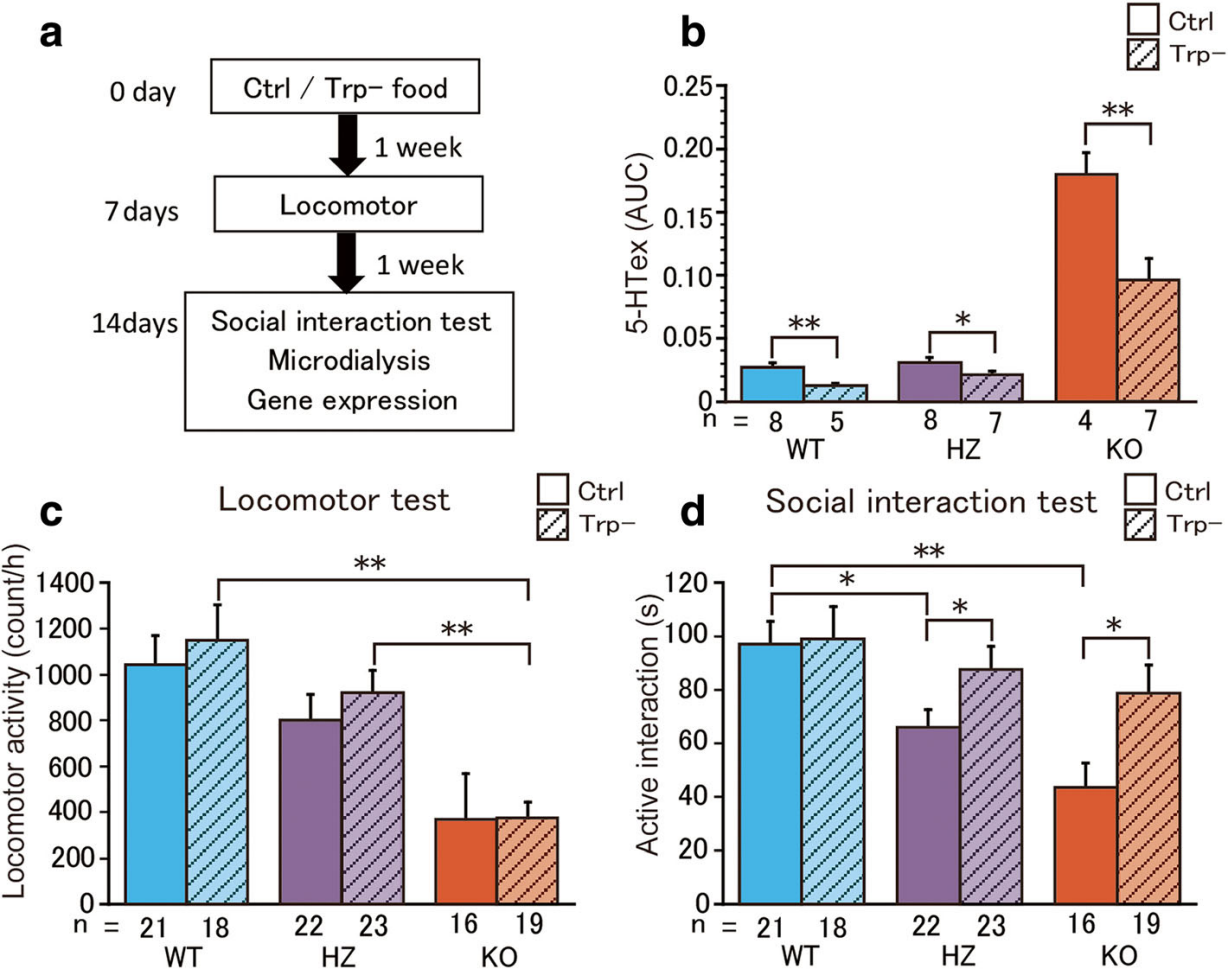
Recovery of behavioral abnormalities by dietary tryptophan depletion in *Sert* HZ and KO mice. **a** Experimental protocol. **b** Extracellular 5-HT levels in the striatum. **c** Locomotor activity over 60 min in the open field test. **d** Active interaction time over 10 min in the social interaction test. The data are expressed as mean ± SEM. *P < 0.05, **P < 0.01, Welch’s *t* test (b). *P < 0.05, **P < 0.01, two-way ANOVA followed by Fisher’s PLSD test (**c**, **d**)

The pathogenetic link between brain 5-HT dysfunction and ASD-relevant social deficits was then investigated by feeding the mice a tryptophan-free diet for 14 days and analyzing behavior and 5-HT levels in the brain (Fig. 3a). Brain 5-HT levels were affected by genotype (*F*_2,33_ = 104.45, *P* < 0.001) and diet (*F*_1,33_ = 24.83, *P* < 0.001), with a significant genotype × diet interaction (*F*_2,33_ = 10.17, *P* < 0.001). Baseline 5-HT levels in the striatum were significantly higher in *Sert* KO mice than in WT and *Sert* HZ mice (*t*_10_ = − 12.80, *P* < 0.001 [WT vs. KO]; *t*_10_ = − 12.16, *P* < 0.001 [HZ vs. KO] by unpaired *t* test; Fig. 3b). *Sert* HZ mice exhibited ASD-like social deficits, but basal 5-HT levels were comparable between WT and *Sert* HZ mice (*t*_14_ = − 0.86, *P* = 0.39 by unpaired *t* test; Fig. 3b). The tryptophan-free diet significantly reduced basal extracellular 5-HT levels in the striatum compared with the control diet in all genotypes (*t*_11_ = 4.23, *P* = 0.0013 [WT]; *t*_13_ = 2.29, *P* = 0.039 [HZ], *t*_9_ = 3.33, *P* = 0.0087 [KO] by unpaired *t* test; Fig. 3b).

Locomotor activity assessed after 7 days on the diet was affected by genotype (*F*_2,113_ = 16.63, *P* < 0.001; Fig. 3c) but not by a tryptophan-free diet (*F*_1,113_ = 0.59, *P* = 0.44; Fig. 3c), with no genotype × diet interaction (*F*_2,113_ = 0.12, *P* = 0.88; Fig. 3c). By contrast, in the social interaction test assessed after 14 days on the diet (Fig. 3d), active interaction was affected by genotype (*F*_2,113_ = 8.04, *P* < 0.001) and diet (*F*_1,113_ = 7.05, *P* = 0.0091), but there was no interaction between genotype and diet (*F*_2,113_ = 1.60, *P* = 0.20). *Sert* HZ and KO mice fed the control diet had deficient social interaction, replicating the results from the first experiment (*P* = 0.012 [WT vs. HZ], *P* < 0.001 [WT vs. KO], *P* = 0.086 [HZ vs. KO] by Fisher’s PLSD post hoc test). The tryptophan-free diet normalized social interaction in *Sert* HZ and KO mice, which were not different from WT mice, exhibiting comparable levels of active social interaction (*P* = 0.35 [WT vs. HZ], *P* = 0.12 [WT vs. KO], *P* = 0.46 [HZ vs. KO] by Fisher’s PLSD post hoc test). The tryptophan-free diet increased social interaction in *Sert* HZ and KO mice (*t*_43_ = − 2.02, *P* = 0.049 [HZ], *t*_33_ = − 2.46, *P* = 0.018 [KO] by unpaired *t* test), whereas social behavior was unaffected in WT mice (*t*_37_ = − 0.15, *P* = 0.88 by unpaired *t* test). These results indicate that the dietary depletion of tryptophan in adulthood reduces extracellular 5-HT levels and alleviates ASD-relevant social deficits in *Sert* mutant mice.

### Gene expression profiles are significantly different between *Sert* HZ and KO mice

**Fig. 4:**
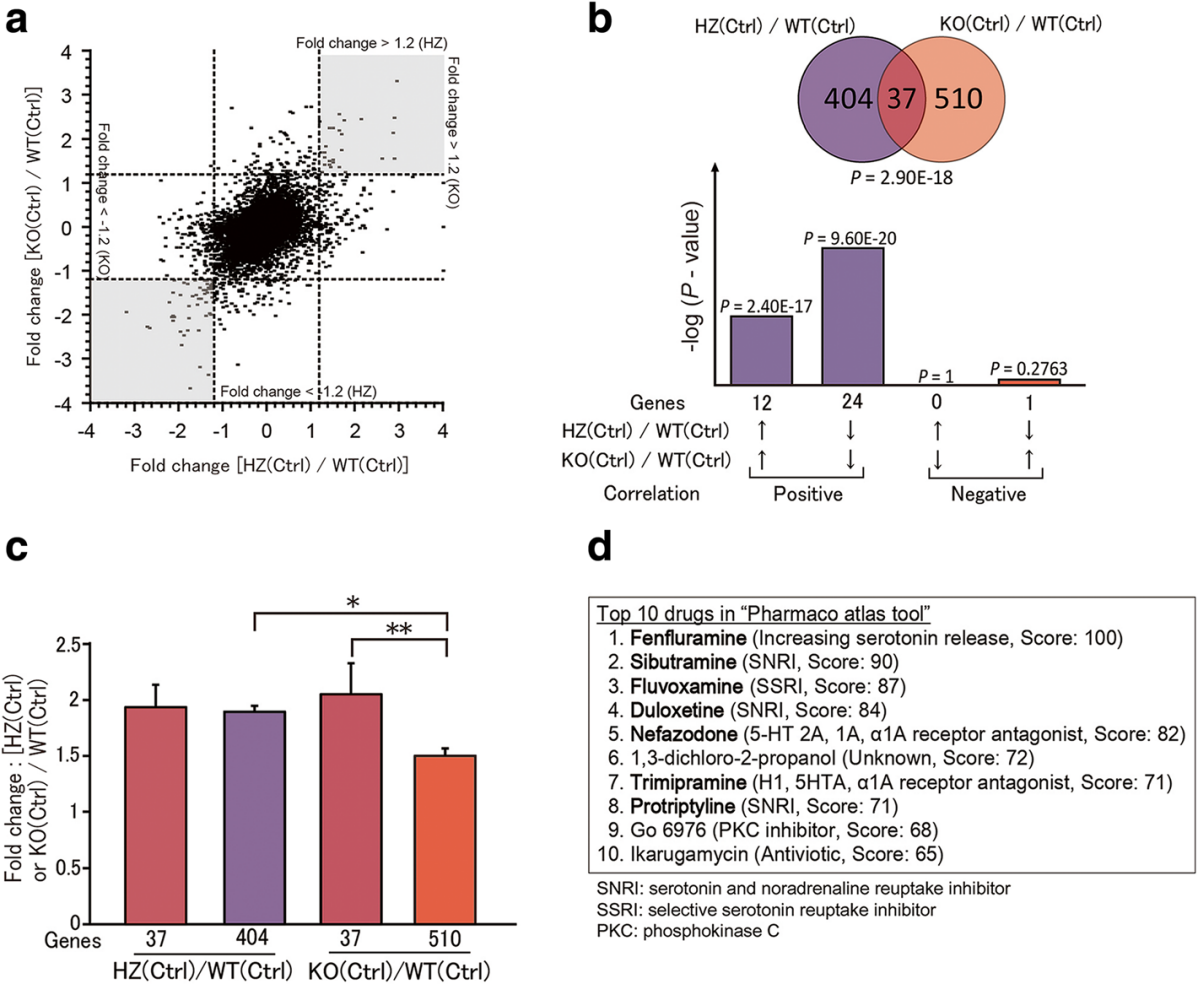
Gene expression changes in the brain in *Sert* HZ and KO mice that received a normal diet. Whole-genome gene expression profiles in whole brains were compared between *Sert* HZ and KO mice. **a** Scatter plot showing the relationship of gene expression changes in *Sert* HZ (HZ[Ctrl]) and KO (KO[Ctrl]) mice compared with WT (WT[Ctrl]) mice. **b** Number of genes with significant changes in levels of expression, showing the correlation of 37 genes whose expression levels were changed in both HZ(Ctrl) and KO(Ctrl) mice. **c** Expression levels in the three groups of genes in Fig. 3b relative to WT(Ctrl). **d** Top 10 drugs with gene expression profiles that were similar to the 37 genes in Fig. 3b. Bold, drugs related to 5-HT signaling

To elucidate the mechanisms that underlie ASD-relevant social deficits and the effects of the tryptophan-free diet in *Sert* HZ and KO mice, whole-genome gene expression profiles were analyzed in *Sert* HZ (Ctrl) and KO (Ctrl) mice. We used whole mouse brains in gene expression analyses of these gene expression profiles. Compared with WT (Ctrl) mice, the fold changes in gene expression were correlated between *Sert* HZ (Ctrl) and KO (Ctrl) mice (*R* = 0.438, *P* < 0.001; Fig. 4a). More genes were significantly changed in *Sert* KO mice than in *Sert* HZ mice. Absolute fold changes that were > 1.2 were obtained for 441 genes between WT (Ctrl) and *Sert* HZ (Ctrl) mice and 547 genes between WT (Ctrl) and *Sert* KO (Ctrl) mice (Fig. 4b, Additional file 2). However, only 37 genes were found to be common between these gene sets, including 12 genes that were upregulated (*P* < 0.001) and 24 genes that were downregulated (*P* < 0.001) in both groups (Fig. 4b, Additional file 1: Table S3). Fold changes in gene expression levels were higher in these 37 genes than in the other 510 genes that were altered in *Sert* KO (Ctrl) mice (*F*_3,986_ = 8.03, *P* < 0.001, Fig. 4c). The Pharmaco Atlas is a gene ontology tool in BaseSpace that is used to search for drugs that are related to alterations of gene expression. The Pharmaco Atlas search revealed that the top 10 candidate drugs regulating the genes affected in both *Sert* HZ and KO mice included at least seven 5-HT-related drugs (Fig. 4d). Common gene expression changes were thus related to 5-HT signal transduction in both *Sert* HZ (Ctrl) and KO (Ctrl) mice despite the overall gene expression profiles being substantially different.

**Fig. 5:**
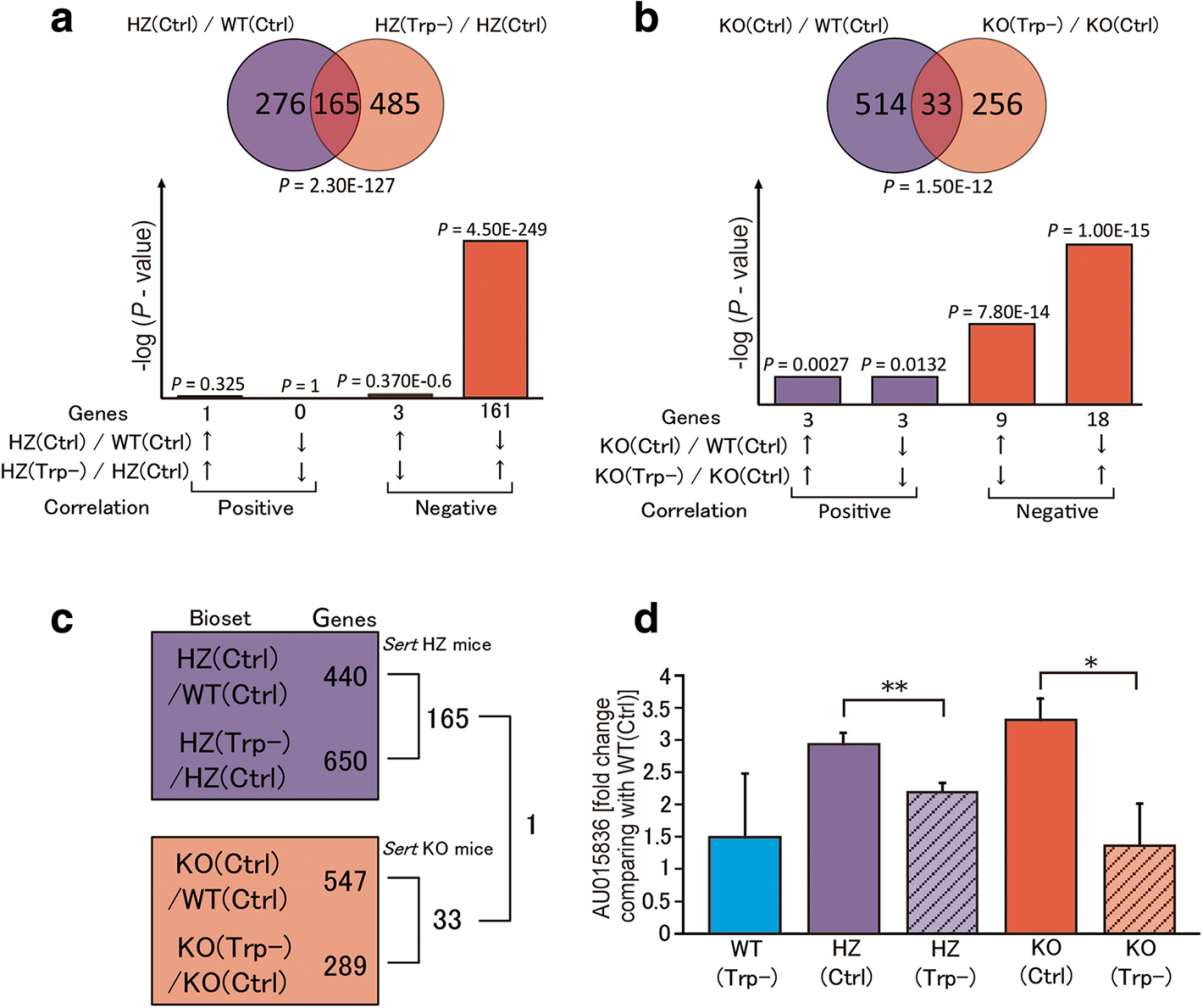
Gene expression changes corresponding to abnormal social behavior in *Sert* HZ and KO mice. **a** Gene expression changes in *Sert* HZ mice that were fed a control diet (HZ[Ctrl]) and tryptophan-free diet (HZ[Trp]), showing the correlation of 165 genes that were commonly altered in both conditions. **b** Gene expression changes in *Sert* KO mice that were fed a control diet (KO[Ctrl]) and tryptophan-free diet (KO[Trp]), showing the correlation of 33 genes that were commonly altered in both conditions. **c** Relationship of genes found in Fig. 4a, b, identifying only one gene that was common to both *Sert* HZ and KO mice. **d** Level of *AU015836* gene expression in *Sert* HZ and KO mice compared with WT(Ctrl) mice. The data are expressed as mean ± SEM. *P < 0.05, **P < 0.01, paired Student’s *t* test

We then sought to identify candidate genes that may be involved in the impairments in social behaviors in *Sert* HZ and KO mice. Gene expression profiles were compared between *Sert* HZ and KO mice that received the tryptophan-free or Ctrl diet. A total of 165 genes were shared between the sets of 441 and 650 genes which were altered between WT (Ctrl) and *Sert* HZ (Ctrl) mice and between *Sert* HZ (Ctrl) and HZ (Trp−) mice (Fig. 5a, Additional file 1: Table S4, Additional file 2). The analysis of *Sert* KO mice indicated a total of 33 genes in common between the sets of 547 and 289 genes which were altered between WT (Ctrl) and *Sert* KO (Ctrl) mice and between *Sert* KO (Ctrl) and KO (Trp−) mice (Fig. 5b, Additional file 1: Table S5). Figure 4c shows the overlap between the following pairs: WT (Ctrl) and *Sert* HZ (Ctrl), HZ (Ctrl) and HZ (Trp−), WT (Ctrl) and KO (Ctrl), and KO (Ctrl) and KO (Trp−). Interestingly, we found that only the expression of *AU015836* was increased in both *Sert* HZ (Ctrl) and KO (Ctrl) mice and normalized with the tryptophan-free diet (*P* = 0.037[KO] and *P* = 0.016[HZ]; Fig. 5d).

### Signaling pathway changes common to *Sert* HZ and KO mice

**Fig. 6:**
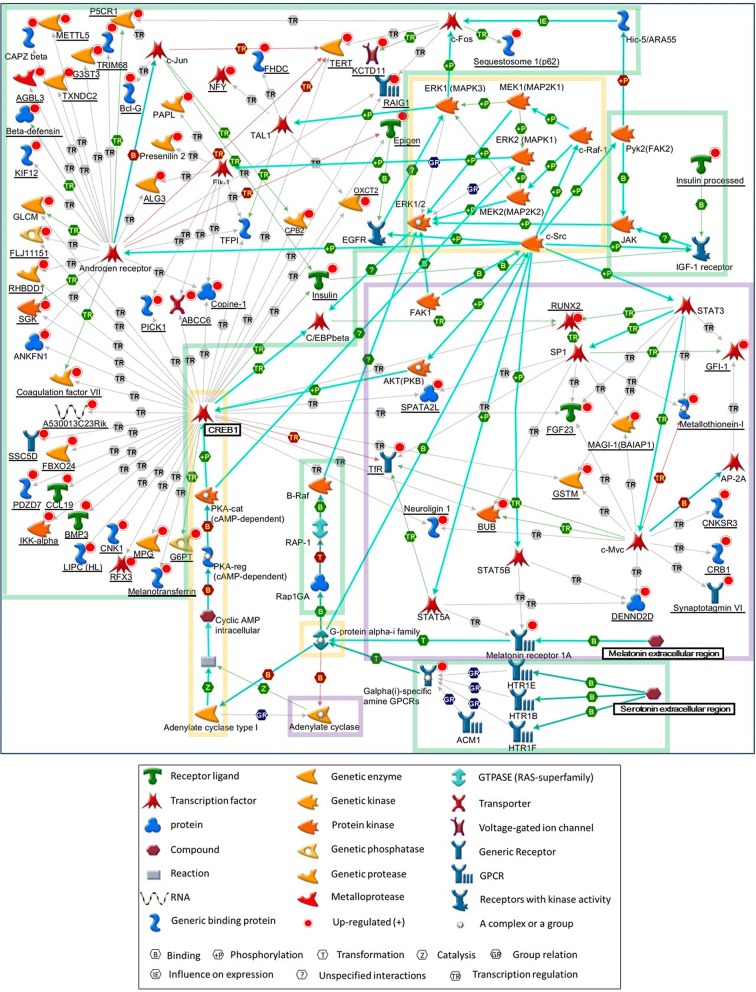
Signaling pathway associated with gene expression changes in *Sert* HZ and KO mice. Diagram of signal transduction pathway associated with genotype and the tryptophan-free diet in *Sert* HZ and KO mice. Green, “CREB1, Elk-1, TAL1, ARIX, p27KIP1-hz2” pathway that contains genes that are affected by tryptophan deficiency in *Sert* HZ mice. Purple, “CREB1, Elk-1, B4GT1, PIP, beta-casein-KO” pathway that contains genes that are affected by tryptophan deficiency in *Sert* KO mice. Orange, genes common to the two pathways. Genes that were included in our data are underlined (*Sert* HZ mice: 41 genes; *Sert* KO mice: 14 genes). The cyan arrows indicate canonical pathways, as recorded by MetaCore. The arrows of each color show the following in the corresponding protein; green, activation; red, inhibition; gray, unspecified

Pathway analysis supported the idea that the gene expression changes in *Sert* HZ and KO mice were different, although overlapping. Pathway analysis using MetaCore was performed to examine alterations of the expression of 165 genes in *Sert* HZ mice that were related to tryptophan depletion. The analysis identified the CREB1/Elk-1/TAL1/ARIX/p27KIP1-hz2 pathway (93 total nodes, 41 seed nodes, *P* < 0.001). In *Sert* KO mice, the 33 genes that were associated with tryptophan depletion were enriched in the CREB1/Elk-1/B4GT1/PIP/beta-casein pathway (69 total nodes, 14 seed nodes, *P* < 0.001). The two pathways partially overlapped (Fig. 6). Most of the genes were under the regulation of transcription factors in the terminal end of the pathway model. The most upstream components were genes associated with extracellular 5-HT function in *Sert* HZ mice and melatonin extracellular function in *Sert* KO mice (Fig. 6). Melatonin is synthesized from tryptophan via 5-HT. These data suggest that high levels of extracellular 5-HT affect gene expression but may do so in a graded fashion, resulting in ASD-relevant social deficits even at lower levels of extracellular 5-HT (*Sert* HZ mice) but producing further changes in function, including alterations of melatonin function, at higher levels of extracellular 5-HT (*Sert* KO mice).

## Discussion

The broad behavioral deficits of *Sert* KO mice that include anxiety-like behavior and hypoactivity [38, 40–42] have discouraged investigators from pursuing *Sert* KO mice because of the lack of selective phenotypes that are characteristic of ASD. Behavioral analyses of *Sert* HZ and KO mice revealed deficits in social interaction in both genotypes. The extreme behavioral abnormalities in *Sert* KO mice might be considered to potentially confound a more specific social phenotype, but this phenotype was apparent in *Sert* HZ mice in the absence of these other features. Additionally, female *Sert* HZ mice exhibited slightly more apparent ASD-relevant social deficits than male *Sert* HZ mice. In humans, ASD symptoms are often thought to be more common in males, but this may result primarily from differences in diagnosis, presentation, or compensation [43]. Nonetheless, our findings support the notion that HZ *Sert* deletion is sufficient to produce ASD-relevant social deficits in both males and females.

Extracellular striatal 5-HT levels were significantly elevated only in *Sert* KO mice and were comparable between WT mice and *Sert* HZ mice, under the conditions examined here. More subtle deficits certainly exist in *Sert* HZ mice [16]. Dietary tryptophan restriction in adulthood lowered striatal 5-HT levels in all three genotypes and normalized ASD-relevant social deficits in both *Sert* HZ and KO mice, whereas the behavior of WT mice was unaffected. Tryptophan deficiency reduces brain 5-HT levels in both humans [44] and rodents [45, 46] and shows beneficial effects in other ASD models. This diet helped to normalize social interactions when given acutely in 129S and C57 mice [47] or chronically in BALB/c mice [45] that have reduced 5-HT function compared to other strains. This evidence supports the present results in *Sert* mutant mice, implicating 5-HT pathways in ASD-relevant social deficits. Although one previous study in humans found that tryptophan depletion exacerbates some ASD deficits [31], in particular repetitive behavior and anxiety, no obvious behavioral alterations of this type were observed in the result from tryptophan depletion in this study in either WT or mutant mice. The fact that reducing tryptophan in adulthood rather than during development ameliorates ASD-relevant social deficits in these mice is a crucial point. There are certainly compensatory changes that occur in *Sert* HZ, and especially KO, mice [48]. The fact that tryptophan depletion is beneficial in adulthood suggests that there is not a developmental window for correcting these deficits, raising the possibility that older children and adults with ASD might benefit from treatments targeting the same serotoninergic dysfunctions, either through altering dietary tryptophan or through other approaches. These findings certainly encourage further exploration of tryptophan depletion in ASD. The influence of tryptophan depletion on other abnormal behaviors, such as in a previous study that reported exacerbated ASD symptoms in humans [31], was not evaluated in the present study. Such behavioral changes as hypoactivity and enhanced anxiety-like behavior are unlikely to result in ASD-relevant social deficits. Future studies that analyze the effects of tryptophan depletion on these behavioral aspects will deepen our understanding of the mechanism of tryptophan depletion.

Comparisons of the gene expression profiles associated with *Sert* genotype and the tryptophan intervention found quite different profiles of gene expression between *Sert* HZ and KO mice, compared to WT mice. Only 8% of the genes altered in *Sert* HZ mice were also altered in *Sert* KO mice. However, the genes that did overlap between these groups all appeared to be also affected by drugs that alter serotonin function. In any case, it would be predicted that if these changes in gene expression were truly associated with the behavioral outcomes that were normalized by the dietary treatment, then the gene expression changes would be reversed. However, only *AU015836* was affected by *Sert* deletion and tryptophan depletion in both *Sert* HZ and KO mice. *AU015836* is encoded on the X chromosome and mainly expressed in the placenta and testis in mice, but the function of *AU015836* remains unknown. This will be of especial interest for this phenotype.

There were also interesting patterns of gene activation in both *Sert* genotype, with and without tryptophan depletion. The analysis of gene expression profiles after tryptophan depletion in *Sert* mutant mice revealed similarities to the influence of SSRIs (Fig. 4d). Evidence suggests that prenatal exposure to SSRIs increases the risk of ASD in humans [49, 50]. However, the behavioral effects of prenatal exposure to SSRIs are controversial in mice and rats [51–53]. There may be critical periods during which the developing brain is particularly vulnerable to elevated 5-HT function that are not addressed by reductions in *Sert* expression here that occurred throughout the lifespan. Nevertheless, since the effects of tryptophan were present in adulthood, this suggests that reversing developmental changes in 5-HT function in ASD may not be necessary to produce positive behavioral outcomes. While SSRIs are effective for some ASD patients [54, 55], SSRI administration during pregnancy is a risk of ASD in the offspring [49, 50]. This contradiction suggests that SSRI treatment may be effective for ASD patients with low levels of extracellular 5-HT. Improvements in ASD-like behavior in 15q11-13 CNV mice by increasing 5-HT levels [56] bolster this hypothesis. On the other hand, our data shows that the reducing 5-HT levels by tryptophan depletion may ameliorate aberrant social behavior caused by increased 5-HT function in *Sert* mutant mice. Perhaps this indicates that both low and high serotonin function may contribute to ASD-relevant social deficits and that a proper balance of 5-HT function is necessary to normalize behavior.

In the present study, the signaling pathway initiated by CREB1 was affected by tryptophan depletion in both *Sert* HZ and KO mice. CREB1 is a transcription factor involved in memory, cognition, and cognitive decline in aging [57–59]. Individuals with a 2q33.4-q34 interstitial deletion have ASD and other symptoms observed in Rett syndrome, and this deletion includes CREB1 [60]. The CREB1 pathway may link abnormally high levels of 5-HT to the development of cellular and circuit-level pathology in ASD. The pathway identified in gene expression profiles was related to 5-HT function in *Sert* HZ mice, whereas this included melatonin function in *Sert* KO mice (biochemically downstream of 5-HT synthesis). Some ASD patients exhibit a decrease in melatonin [61, 62] and an aberrant 5-HT-melatonin pathway [63]. Research that focuses on melatonin in *Sert* mutant mice will provide additional insights into the role of melatonin in ASD-like, and other comorbid, deficits.

Comparisons of *Sert* HZ and KO mice helped overcome one of the basic limitations of using solely *Sert* KO mice as a disease model to study ASD. *Sert* KO mice have other non-ASD-like phenotypes and complete SERT deletions are not seen in humans. This is similar to the situation with dopamine transporter (DAT) KO mice [64] that have been proposed to be an animal model of schizophrenia and attention-deficit/hyperactivity disorder (ADHD) [65–67]. Although these mice have phenotypes characteristic of these conditions in many respects, humans with a complete loss of DAT expression are very rare and develop infantile parkinsonism-dystonia, a devastating, and ultimately lethal, movement disorder [68]. However, reduced DAT expression in the brain is observed in patients with schizophrenia [69] and ADHD [70]. Consistent with this, DAT HZ mice display some mild ADHD-like phenotypes, although not phenotypes that are related to schizophrenia or bipolar disorder [71]. Similarly, lower SERT expression is associated with ASD in humans [15, 72], and *Sert* HZ mice exhibit ASD-relevant social deficits without other behavioral abnormalities. One limitation of the present study is that the influence of 2 weeks of tryptophan depletion on locomotor activity was not quantitatively evaluated. Therefore, unclear is whether 2 weeks of tryptophan depletion increased locomotor activity so as to help the recovery of social interaction in *Sert* KO mice. Nonetheless, investigations of *Sert* HZ mice may contribute to bridging the gap between ASD in humans and mouse models of ASD, and treatments that normalize 5-HT function may be potential treatments for at least some individuals with ASD.

## Conclusions

The present study identified an important causal link between a reduction of *Sert* function and ASD-relevant social deficits in mice. Moreover, the production of this behavior requires only HZ deletion of *Sert.* The critical role of 5-HT is confirmed by the finding that tryptophan depletion in adulthood improves social interaction and decreases extracellular 5-HT levels in the striatum. This finding raises hope for similar approaches to help ASD patients. A potential underlying pathological mechanism of ASD-relevant social deficits was found at the level of gene expression. The *AU015836* gene was the only gene found to change in both *Sert* HZ and KO mice and to be reversed by altering dietary tryptophan intake. However, a broader set of signaling pathways were also implicated that were highly similar to SSRI-related gene sets, including CREB1-related signaling pathways. These results will contribute to a better understanding of the pathophysiology of ASD, the role of altered 5-HT function in ASD, and may ultimately help in the development of novel therapeutic approaches for the treatment of ASD.

## Additional files

Additional file 1: Protocol on behavioral tests and lists of gene expression in detail. (DOCX 79 kb)

Additional file 2: Lists of the genes analyzed in Fig. 5. (XLSX 63 kb)

## Abbreviations

ADHD: Attention-deficit/hyperactivity disorder
ASD: Autism spectrum disorder
CREB1: cAMP response element-binding protein 1
DAT: Dopamine transporter
HZ: Heterozygous
KO: Knockout
PIP: Prolactin-induced protein
SERT: Serotonin transporter
TAL1: T cell acute lymphocytic leukemia 1
TPH: Tryptophan hydroxylase
Trp−: Tryptophan-free diet
WT: Wild type
