# Supplementary material for: Brain hyperserotonemia causes autism-relevant social deficits in mice
Source: Mol Autism. 2018 Nov 26;9:60. doi: 10.1186/s13229-018-0243-3 (PMC6258166; doi:10.1186/s13229-018-0243-3)
Supplement: Supplementary file 1 — Protocol on behavioral tests and lists of gene expression in detail. (DOCX 79 kb) [file 13229_2018_243_MOESM1_ESM.docx]

Additional file 1

Table S1. Experimental protocol.

Table S2. The list of used mice in gene expression analysis.

Table S3. Overlapped genes differentially expressed in both *Sert* KO and HZ data sets, as determined by microarray analysis. Blue indicates gene upregulations and red indicates gene downregulations.

Table S4. Overlapped genes differentially expressed in both Trp- data sets and Ctrl data sets in *Sert* HZ, as determined by microarray analysis.

Table S5. Overlapped genes differentially expressed in both Trp- data sets and Ctrl data sets in *Sert* KO, as determined by microarray analysis. Blue indicates gene upregulations and red indicates gene downregulations.
